# The use of the tyrosine kinase inhibitor Nilotinib in Spondyloarthritis: does targeting inflammatory pathways with a treatment lead to vascular toxicity?

**DOI:** 10.1186/s12967-017-1334-1

**Published:** 2017-12-15

**Authors:** Loukman Omarjee, Vincent Jaquinandi, Guillaume Mahe

**Affiliations:** 1 Vascular Medicine Department, CHU de Rennes, 35033 Rennes Cedex 9, France; 2 Univ Rennes, CHU Rennes, INSERM, CIC 1414, 35000 Rennes, France; 3 Department of Vascular Medicine, Centre Hospitalier de Redon, 35600 Redon, France; 4 Vascular Medicine Department, Pôle imagerie médicale et explorations fonctionnelles, Hôpital Pontchaillou, CHU de Rennes, 2 rue Henri Le Guilloux, Rennes, 35033 France

**Keywords:** Spondyloarthritis, Nilotinib, Tyrosine kinase inhibitor, Vascular toxicity, Major adverse cardiovascular events, Peripheral artery disease, Inflammation, Atherosclerosis, Morbi-mortality

## Abstract

Spondylarthritis (SpA) is an inflammatory rheumatic disease associated with increased incidence of major adverse cardiovascular events (MACEs). Recently, Paramarta et al. proposed the use of the tyrosine kinase inhibitor Nilotinib in Spondyloarthritis to target certain inflammatory pathways. However, Nilotinib, which is highly effective for the treatment of patients with chronic myeloid leukaemia (CML), is also associated with an increased risk of MACEs. The authors suggest that Nilotinib may be effective in peripheral SpA by modulating inflammation, but not in axial SpA. Considering the vascular toxicity of Nilotinib and the acceleration of atherosclerosis in SpA patients, we suggest taking MACEs as an end-point in future trials.

## Background

Spondylarthritis (SpA) is a chronic inflammatory rheumatic disease that can result in significant disability [1]. It is associated with increased incidence of major adverse cardiovascular events (MACEs) [2]. With the emergence of Tumor Necrosis Factor Inhibitors (TNFi), such as Infliximab^®^, Etanercept^®^, Adalimumab^®^ or Cetolizumab^®^, therapeutic outcomes in SpA have improved substantially [3]. However, there is still an unmet need for a subset of patients who do not respond adequately to TNFi [3]. New biological molecules blocking extra-cellular cytokines involved in new pathways of inflammation such as IL-17 (Secukinumab^®^) and IL-23 (Ustekinumab^®^) inhibitors showed their effectiveness in psoriasis, psoriatic arthritis and SpA [4]. Targeting the production of intracellular cytokines by synthetic small molecules such as Janus Kinase (JAK) Inhibitor (Tofacitinib^®^), Phosphodiesterase-4 (PDE-4) Inhibitor (Apremilast^®^) or Tyrosine Kinase (TK) Inhibitor (Imatinib^®^, Nilotinib^®^) is a growing field. The later, originally developed to inhibit BCR-ABL in Chronic Myeloid Leukemia (CML), could also inhibit c-KIT, the receptor for stem cell factor, thereby inducing apoptosis of mast cells, including synovial mast cells involved in inflammatory pathways [5]. However, Nilotinib, which is highly effective for the treatment of patients with CML, it is associated with an increased risk of MACEs [6]. In this review, we will discuss the concept of accelerated atherosclerosis in SpA and the vascular toxicity of Nilotinib.

## Main text

Recently, Paramarta et al. published “A proof-of-concept study with the tyrosine kinase inhibitor Nilotinib in Spondyloarthritis” [1]. However, an acceleration of the atherosclerosis process leading to major adverse cardiovascular events (MACEs) in Spondylarthritis (SpA) has been reported [2]. Nilotinib, which is highly effective for the treatment of patients with CML, is associated with an increased risk of MACEs [6]. Therefore it is questionable to use Nilotinib in SpA patients. In what follows, we present a review on SpA and Nilotinib cardiovascular involvement:

1. SpA is a systemic autoimmune inflammatory rheumatic disease affecting the axial and/or peripheral skeleton [1]. A population-based study showed that SpA patients had an increased incidence of cardiovascular (CV) disease [2]. The association between SpA and CV risk should be investigated according the European Society of Cardiology guidelines, which have a specific section dedicated to preventing CV disease in patients with systemic autoimmune inflammatory diseases [7]. Furthermore, an increase in CV mortality among SpA patients has been reported in several studies [8]. In one of them, 677 patients with SpA were followed over a period of 35 years [8]. The mortality rate in the SpA group was 14.5% in this study [8]. CV diseases are the leading cause of death (40%), followed by cancer (26.8%) and infections (23.2%) [8]. Compared to a control population matched for age, gender and geographic area, the survival rate was significantly lower in the SpA group [8]. In addition, an increase in CV morbidity was also found for the SpA patients [2, 9]. Two large-scale epidemiological studies have been conducted, one in Canada and the other in Sweden [2, 9]. The Canadian study showed an increase in the incidence of ischemic heart disease by 37% and stroke by 25% compared to the general population [2]. The Swedish study also showed a significant increase in the incidence of ischemic stroke with an estimated risk of 2.02 (95% confidence interval [95% CI] 1.90–2.14) [9]. One meta-analysis found a significant increase in myocardial infarction risk (MI) of 60% among 17,903 patients compared to 1,300,000 controls (OR = 1.60 [95% CI 1.32–1.93]). Similarly, in another study, the risk of stroke was increased by 50% in the SpA group (9791 patients) compared to the control group (1,239,041 controls) (OR = 1.50 [95% CI 1.39–1. 62]) [10]. Finally, the risk of PAD was increased by 13.5% in one study on SpA patients [11].
2. This increase in CV morbi-mortality can be linked to inflammation. Inflammation is at the cornerstone of the process, generating endothelial lesions and dysfunctions leading to atherosclerosis. But inflammation also amplifies the disease process arising from the classic CV risk factors [12]. Furthermore, in one study, values for markers of oxidative stress, lipid profile, and inflammation, as well as soluble CD40 ligand (sCD40L), placental growth factor (PlGF), and carotid Intima–Media Thickness (IMT) were significantly higher in the SpA group compared to the healthy group, leading to an increased risk for atherosclerosis [13].
3. The use of synthetic and biological disease-modifying anti-rheumatic drugs (DMARDs) has completely changed the natural history of these systemic autoimmune inflammatory diseases by targeting different pathways leading to chronic inflammation [14]. Data on the use of these drugs suggests favourable effects on CV risk in patients with rheumatoid arthritis and psoriatic arthritis [15]. However, the use of corticosteroid therapy is associated with unfavourable CV and metabolic effects in SpA patients [16]. On the other hand, in atherosclerotic patients without systemic autoimmune inflammatory disease and with previous MI, the Canakinumab^®^ Anti-inflammatory Thrombosis Outcome Study (CANTOS) showed that therapy targeting the inflammatory cytokine interleukin-1β innate immunity pathway with Canakinumab^®^ led to a significantly lower rate of recurrent cardiovascular events than a placebo, independently from the lowering of lipid levels [17].
4. Finding new DMARDs in SpA is a daily challenge for several research teams worldwide. One therapeutic target is the use of protein tyrosine kinase inhibitors (PTKIs) as suggested by Paramarta et al. [1]. These small molecules have revolutionized the treatment and outcomes of CML, changing it from a life-threatening disease to one with a life expectancy similar to the general population for patients who are responsive to treatment [18–20]. Protein tyrosine kinases (PTKs) are enzymes that catalyse the transfer of phosphate from adenosine triphosphate (ATP) to tyrosine residues on specific proteins [21] and play a critical role in vascular, metabolic and myocardial biology and physiology [22]. All approved TKIs for the treatment of CML target the BCR–ABL protein with TK activity but they also possess different effects on other kinases, including those involved in the cardiovascular system, such as Platelet-derived growth factor receptors (PDGFRs) which can lead to CV toxic effects [6]. Nilotinib^®^ is a highly effective TKI in the treatment of CML. However, reports of CV toxicity caused by Nilotinib^®^ have recently raised concern [23]. Reports of PAD and MACEs in patients exposed to Nilotinib^®^ are increasingly reported. The first concerns about the vascular toxicity of Nilotinib^®^ were reported for 3 patients under treatment who developed PAD [23]. In a retrospective analysis of 179 patients, 12 patients (6.2%) developed PAD involving their lower limbs, 8 patients required invasive therapy, such as angioplasty and stenting, and 4 patients required amputation [24]. Unfortunately, PAD was also found not only in patients in whom CV risk factors were present, but sometimes also in younger patients without any risk factors [23]. In addition to PAD, Nilotinib^®^-treated patients can develop cerebral ischemia and MI [23]. Further to this, data from the Food and Drug Administration Adverse Event Reporting System (FARES) shows an increase in coronary artery stenosis and angina pectoris after 1 year of Nilotinib^®^ in CML patients; MI and PAD after 2 years; femoral arterial stenosis and intermittent claudication after 3 years; and acute coronary syndrome, peripheral ischemia and femoral arterial occlusion after 4 years [25]. In a prospective study including 159 CML patients treated with either Imatinib^®^ or Nilotinib^®^, PAD defined by an abnormal ankle–brachial index (ABI) was more prevalent among patients treated with Nilotinib than among patients on Imatinib^®^[26]. The atherothrombotic vascular events occurring in some Nilotinib^®^-treated patients could arise from a combination of genetic and biochemical factors, such as increased lipid peroxidation due to detrimental Lipoxygenase-1 (LOX-1) polymorphism and an imbalance in cytokine-driven inflammation, mainly brought about by the strong reduction in anti-inflammatory cytokine IL-10 levels [27]. Furthermore, Nilotinib^®^ is associated with several metabolic disturbances, including hyperglycemia, perhaps via insulin resistance, and dyslipidemia, which can develop within less than 3 months of treatment. However, these metabolic disturbances certainly do not explain all cases of vascular adverse events, and are probably among the many contributory mechanisms. In a mouse model of hind limb ischemia, Nilotinib^®^ was found to slow blood flow recovery after the induction of ischemia, which was accompanied by an increased levels of limb necrosis [28]. According to one report, Nilotinib^®^ was reported to exert direct pro-atherogenic and anti-angiogenic effects on vascular endothelial cells, leading to PAD in patients with CML [28]. Furthermore, Nilotinib^®^ induced a significant depletion of TK cKIT+ mast cells, reported in peripheral SpA patients by these authors. Nilotinib^®^ was shown to promote the expression of pro-atherogenic cytoadhesion molecules (CAMs) on cultured human umbilical vein endothelial cells (HUVEC), including ICAM-1 (CD54), VCAM-1 (CD106) and E-Selectin (CD62E). Using chemical proteomic profiling and phosphor-array analysis, Nilotinib^®^ was shown to bind to several antigenic targets in endothelial cells, including Tie-2/TEK, JAK1, BRAF and EPHB2. In addition, the inhibition of several kinases involved in vascular cell homeostasis, such as DDR1, cKIT, and/or PDGFR, has been suggested as a potential mechanism implicated in Nilotinib^®^-induced vascular events. Finally, CV risk factors, PAD and other atherothrombotic events were screened in 75 patients who had received either Imatinib^®^ (N = 39) or Nilotinib^®^ (N = 36) [29]. Twenty-five per cent of the patients receiving Nilotinib^®^ developed PAD, ACS or stroke, as compared to 7.6% of the patients receiving Imatinib^®^. In this study, Nilotinib^®^-treated patients had an unbalanced pro/anti-inflammatory network. The authors hypothesized that this pro-inflammatory state could cause pro-atherothrombotic activation via enhanced lipid peroxidation, and that genetic pro-atherothrombotic predisposition conferred by LOX-1 could play a role in the increased incidence of vascular events [29].

## Conclusion

To conclude, considering the vascular toxicity of Nilotinib^®^ and accelerated atherosclerosis in SpA patients, we suggest taking MACEs as an end-point in future trials. Finally, patients with systemic autoimmune inflammatory diseases such as SpA should be strictly screened for modifiable risk factors such as obesity, hypertension, dyslipidemia, diabetes, and cigarette smoking, especially under Nilotinib^®^ treatment. The management of such patients should follow guidelines on preventing CV disease in patients with systemic autoimmune inflammatory diseases [7]. In addition, the European LeukaemiaNet recommends PAD screening by ABI or duplex ultrasonography every 6–12 months for patients on Nilotinib [30]. We suggest that authors should take ABI as a safety variable in future trials. In the era of precision medicine and progress in pharmacogenomics, IVS4-14 G/G LOX-1 polymorphism should be investigated before Nilotinib^®^ introduction, insofar as it constitutes the strongest predictive factor for a higher incidence of CV events [27].

## Abbreviations

SpA: Spondyloarthritis
TNFi: Tumor Necrosis Factor Inhibitor
MI: myocardial infarction
PAD: peripheral arterial disease
CI: confidence interval
OR: odds ratio
CML: chronic myeloid leukaemia
FDA: Food and Drug Administration
FARES: FDA Adverse Event Reporting System
CAM: cytoadhesion molecules
HUVEC: human umbilical vein endothelial cells
ICAM-1: intercellular adhesion molecule 1
VCAM-1: vascular cell adhesion molecule 1
E-Selectin: endothelial selectin
JAK1: janus kinase 1
EPHB2: ephrin B2
DDR1: discoidin domain receptor tyrosine kinase 1
PDGFR: platelet-derived growth factor receptors
MACE: major adverse cardiovascular events
CV: cardiovascular
sCD40L: soluble CD40 ligand sCD40L
PlGF: placental growth factor
IMT: Intima–Media Thickness
DMARDs: disease-modifying anti-rheumatic drugs
CANTOS: Canakinumab Anti-inflammatory Thrombosis Outcome Study
TKIs: tyrosine kinase inhibitors
TK: tyrosine kinase
ATP: adenosine triphosphate
